# Epidemiological and genetic analysis of Cetacean Morbillivirus circulating on the Italian coast between 2018 and 2021

**DOI:** 10.3389/fvets.2023.1216838

**Published:** 2023-07-31

**Authors:** Ignacio Vargas-Castro, Simone Peletto, Virginia Mattioda, Maria Goria, Laura Serracca, Katia Varello, José Manuel Sánchez-Vizcaíno, Roberto Puleio, Fabio Di Nocera, Giuseppe Lucifora, Pierluigi Acutis, Cristina Casalone, Carla Grattarola, Federica Giorda

**Affiliations:** ^1^VISAVET Center and Animal Health Department, Veterinary School, Complutense University of Madrid, Madrid, Spain; ^2^Istituto Zooprofilattico Sperimentale del Piemonte, Liguria e Valle d'Aosta - WOAH Collaborating Centre for the Health of Marine Mammals, Turin, Italy; ^3^Istituto Zooprofilattico Sperimentale della Sicilia, Palermo, Italy; ^4^Istituto Zooprofilattico Sperimentale del Mezzogiorno, Naples, Italy

**Keywords:** CeMV, phylogenesis, epidemiology, stranding, Italy, cetaceans, Mediterranean Sea

## Abstract

Cetacean morbillivirus (CeMV) has caused several outbreaks, unusual mortality events, and interepidemic single-lethal disease episodes in the Mediterranean Sea. Since 2012, a new strain with a northeast (NE) Atlantic origin has been circulating among Mediterranean cetaceans, causing numerous deaths. The objective of this study was to determine the prevalence of CeMV in cetaceans stranded in Italy between 2018 and 2021 and characterize the strain of CeMV circulating. Out of the 354 stranded cetaceans along the Italian coastlines, 113 were CeMV-positive. This prevalence (31.9%) is one of the highest reported without an associated outbreak. All marine sectors along the Italian coastlines, except for the northern Adriatic coast, reported a positive molecular diagnosis of CeMV. In one-third of the CeMV-positive cetaceans submitted to a histological evaluation, a chronic form of the infection (detectable viral antigen, the absence of associated lesions, and concomitant coinfections) was suspected. Tissues from 24 animals were used to characterize the strain, obtaining 57 sequences from phosphoprotein, nucleocapsid, and fusion protein genes, which were submitted to GenBank. Our sequences showed the highest identity with NE-Atlantic strain sequences, and in the phylogenetic study, they clustered together with them. Regarding age and species, most of these individuals were adults (17/24, 70.83%) and striped dolphins (19/24, 79.16%). This study improves our understanding on the NE-Atlantic CeMV strain in the Italian waters, supporting the hypothesis of an endemic circulation of the virus in this area; however, additional studies are necessary to deeply comprehend the epidemiology of this strain in the Mediterranean Sea.

## Introduction

Cetacean morbillivirus (CeMV) is an enveloped, negative-strand RNA virus classified into the genus *Morbillivirus* and family *Paramyxoviridae* (1). Two different lineages of CeMV have been described to date: the CeMV-1 lineage, which includes dolphin morbillivirus (DMV), porpoise morbillivirus (PMV), pilot whale morbillivirus (PWMV), and beaked whale morbillivirus (BWMV), and the CeMV-2 lineage, which includes two strains detected in a Guiana dolphin (*Sotalia guianensis*) from Brazil and in an Indo-Pacific bottlenose dolphin (*Tursiops aduncus*) from Australia (2–5).

CeMV has been responsible for several outbreaks, unusual mortality events (UMEs), and interepidemic single-lethal disease episodes (6–9), and it is regarded as the non-anthropogenic agent with the greatest impact on cetacean health and conservation worldwide (10). The first documented CeMV outbreak in the Mediterranean Sea, between 1990 and 1992, killed thousands of striped dolphins (*Stenella coeruleoalba*) (11, 12). Since then, several UMEs associated with DMV, have been reported on the Mediterranean coasts: two of them in Spain during the period of 2006–2008 and in 2011 (13, 14); one in France during the period of 2007–2008 (15); and three in Italy during the period of 2011–2013, in 2014, and in 2016 (16–19).

Epidemiological surveillance and genetic analysis of the detected sequences revealed that, from 2012, a new strain with a northeast (NE) Atlantic origin caused deaths of cetaceans along the Spanish Mediterranean coast (20) and subsequently in Italy: both an UME (18) and isolated cases (21, 22). The systematic sanitary surveillance of cetaceans is a priority for understanding potential infectious disease outbreaks (23) and elucidating the dynamics of the CeMV infection (24). In this study, our primary objectives were to document the prevalence of CeMV in the cetacean populations from the Italian waters and to provide detailed epidemiological data in terms of species, age, and stranding locations. In addition, through the phylogenetic analysis performed, we characterized the CeMV strain circulating.

## Methods

### Animals

All animals were stranded cetaceans examined during routine pathological analysis and cause-of-death assessment by the Italian Cetacean Stranding Network of the Istituti Zooprofilattici Sperimentali, the veterinary public health institutions under the Italian Ministry of Health, and coordinated by the National Reference Center for Diagnostic Investigations in Stranded Marine Mammals (C.Re.Di.Ma). The animals were submitted for a complete *post-mortem* examination according to standard protocols (25).

Between 2018 and 2021, 829 cetacean strandings were reported along the Italian coastlines (26–29). A complete necropsy was performed, when possible (*n* = 376/829; 45.36%), based on the decomposition condition code (DCC), according to standardized protocols (25, 30). Age classes (neonates-calves, juveniles-subadults, and adults) were estimated based on total body length (25, 31). During necropsy, tissue samples from major organs were collected and divided into three aliquots for subsequent analysis: one was kept frozen at −20°C for microbiological analysis, one at −80°C for biomolecular analysis, and the third was preserved in neutral buffered formalin for histological and immunohistochemical (IHC) analysis.

Annual stranding reports elaborated by C.Re.Di.Ma (26–29), referring to the period 2018–2021, were retrieved and further analyzed to get (i) the CeMV prevalence during the study period, (ii) the signaling (species, sex, and age) and epidemiological information (stranding date and location), (iii) the descriptions of the histopathological examination, with a focus on CeMV suggestive lesions (2), and (iv) the results of the ancillary investigations of selected pathogens in CeMV-positive animals.

### CeMV analysis: confirmation and characterization

A total of 134 CeMV target tissues (brain, cerebrospinal fluid, pharyngeal tonsils, lung, mesenteric, pre-scapular and pulmonary lymph nodes, spleen, liver, kidney, urinary bladder, placenta, tongue, and blood plasma, when possible) from 33 cetaceans out of a total of 113 CeMV molecularly positive cetaceans stranded along the Italian coastlines in the period under study were submitted to confirmatory and characterization analysis following Verna et al. (32), and Bellière et al. (33) methodologies, respectively.

Briefly, tissues were physically disrupted using a TissueLyser II homogenizer (Qiagen, Hilden, Germany) by high-speed shaking in plastic tubes with stainless-steel beads (5 mm diameter), and RNA was extracted using an All-Prep DNA/RNA Mini kit (Qiagen, Hilden, Germany), based on the manufacturer's instructions. A molecular diagnosis following Verna et al. (32) and targeting the nucleocapsid (N) gene with the use of degenerate primers. Characterization based on the phosphoprotein (P), N, and fusion protein (F) genes was conducted according to Bellière et al. (33) through various conventional reverse transcription-PCR assays. The N gene was amplified using NgeneF and NgeneR primers, resulting in a 158-bp amplicon. The F gene was amplified with DMVFu-F and DMVFu-R primers, generating a 152-bp amplicon. The P gene was amplified using DMV-C and DMV-P2 primers, producing a 358-bp amplicon. Positive products from the characterization analysis were subsequently sequenced by the Sanger sequencing method.

### Phylogenetic study

Nucleotide identities (1 – p-distance) for the sequences of P and N genes were calculated using the MEGA X software (34).

The CeMV nucleotide sequences were analyzed phylogenetically using the maximum likelihood method in the MEGA X software (34). To generate a reliable phylogenetic tree, we confirmed the accuracy of sequence alignments, since the average amino acid p-distances were 0.08 and 0.03 for P and N genes, respectively. These values were smaller than the acceptance threshold value (< 0.8) for average p-distance (35, 36). The Kimura 2-parameter model was used with 2,000 bootstrap replicates, and the resulting trees were rooted and edited with the iTOL editor (37).

The heterogeneity of the available DMV sequences prevented the maintenance of a consistent set of alignment for each gene, as previously mentioned by other authors (38).

## Results

**Figure 1:**
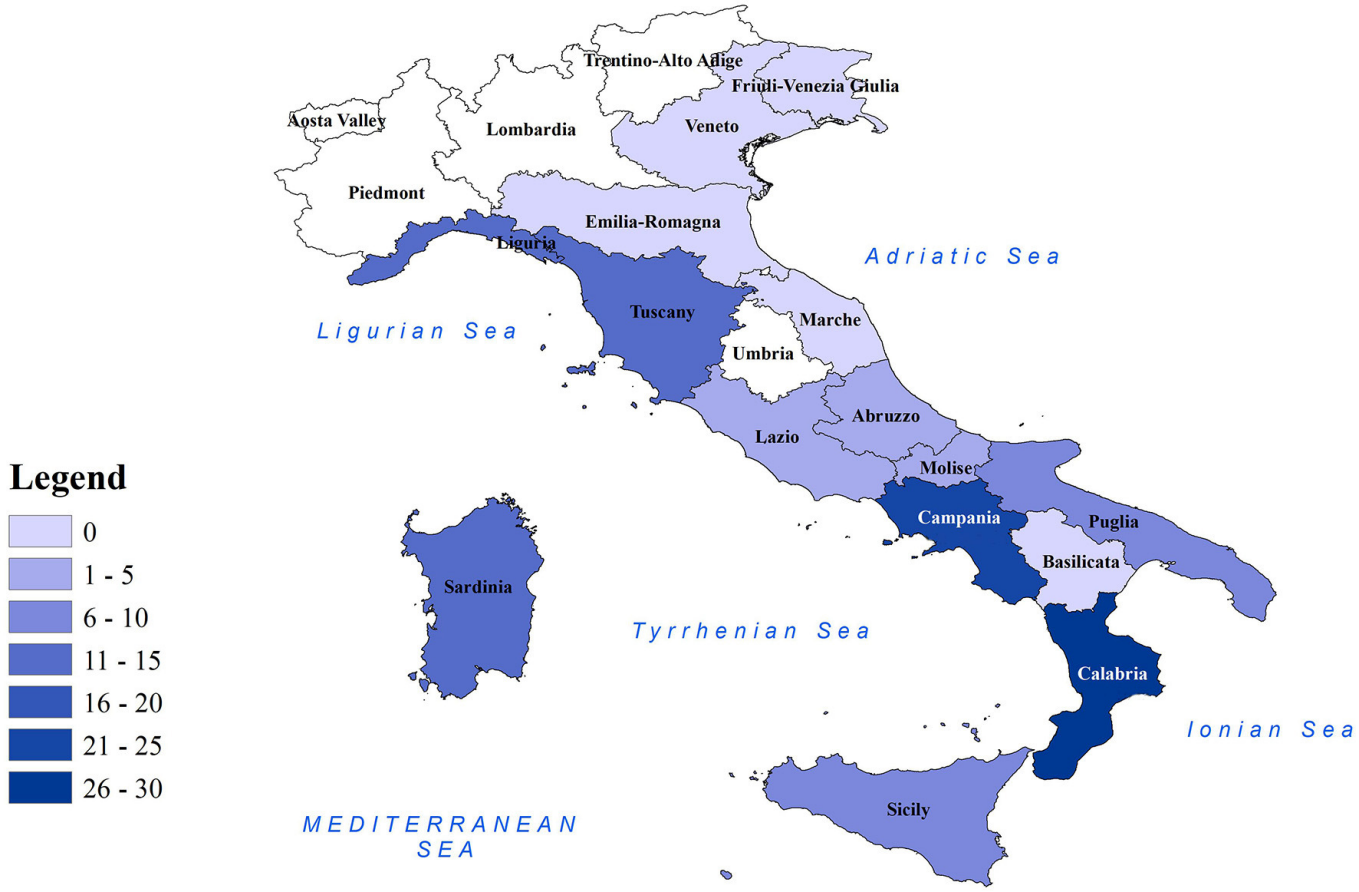
Distribution of CeMV-positive cetaceans in Italian regions. Map of Italian regions color-coded based on the number of CeMV-positive cetaceans found. Coastal regions are depicted in colored shading, while non-coastal regions are shown in white.

Between 2018 and 2021, a total of 1,788 tissues from 354 cetaceans were molecularly screened for CeMV detection by the Italian Stranding Network. These cetaceans were among the 829 stranded along the Italian coasts during the aforementioned period (26–29). In 2018, tissues from 72 cetaceans were analyzed, resulting in a total of 343 tissues tested, with a range of 1–11 tissues per animal and an average of 4.76 tissues per animal. Similarly, in 2019, tissues from 97 cetaceans were examined, resulting in a total of 527 tissues tested, with a range of 1–12 tissues per animal and an average of 5.43 tissues per animal. For the year 2020, tissues from 92 cetaceans were assessed, amounting to 495 tissues tested, with a range of 1–11 tissues per animal and an average of 5.38 tissues per animal. Finally, in 2021, tissues from 93 cetaceans were screened, resulting in a total of 423 tissues tested, with a range of 1–12 tissues per animal and an average of 4.54 tissues per animal. CeMV positivity was detected in 113 specimens (113/354, 31.9%) (Table 1 and Figure 1). The age distribution of these cetaceans was (from highest to lowest percentage) as follows: adults (57/113, 50.44%), juveniles-subadults (47/113, 41.59%), neonates-calves (8/113, 7.08%), and, in one case (1/113, 0.88%), the age could not be determined (Table 1).

**Table 1:** CeMV occurrence data from Italian coast between 2018 and 2021.

**Year**	**Tested/ total stranded**	**Prevalence of CeMV**	**Species of the CeMV-positive cetaceans**	**Age of the CeMV-positive cetaceans**	Lesions compatible with CeMV in CeMV-positive cetaceans^*^	**Regions**	**Reference**
2018	72/174	30.55% (22/72)	*Stenella coeruleoalba*: *n =* 20 *Physeter macrocephalus*: *n =* 2	Juveniles-subadults: *n =* 7 Adults: *n =* 15	*n =* 14 (14/22, 63.63%) Non-suppurative meningoencephalitis (*n =* 12) Bronchointerstitial pneumonia (*n =* 5) Lymphoid depletion (*n =* 4)	Calabria (*n =* 6), Liguria (*n =* 4), Sicily (*n =* 3), Campania (*n =* 3), Sardinia (*n =* 2), Puglia (*n =* 2), Tuscany (*n =* 1), and Lazio (*n =* 1)	(26)
2019	97/240	45.36% (44/97)	*Stenella coeruleoalba*: *n =* 26 *Tursiops truncatus*: *n =* 11 *Physeter macrocephalus*: *n =* 4 *Balaenoptera physalus*: *n =* 1 *Globicephala melas*: *n =* 1 *Ziphius cavirostris*: *n =* 1	Neonates-calves: *n =* 2 Juveniles-subadults: *n =* 21 Adults: *n =* 21	*n =* 19 (19/30, 63.33%) Non-suppurative meningoencephalitis (*n =* 13) Bronchointerstitial pneumonia (*n =* 9) Lymphoid depletion (*n =* 5)	Campania (*n =* 8), Sardinia (*n =* 7), Calabria (*n =* 6), Liguria (*n =* 6), Tuscany (*n =* 5), Puglia (*n =* 3), Lazio (*n =* 3), Sicily (*n =* 3), Abruzzo (*n =* 2), and Molise (*n =* 1)	(27)
2020	92/198	23.91% (22/92)	*Stenella coeruleoalba*: *n =* 20 *Balaenoptera physalus*: *n =* 1 *Grampus griseus*: *n =* 1	Neonates-calves: *n =* 3 Juveniles-subadults: *n =* 11 Adults: *n =* 8	*n =* 12 (12/18, 66.67%) Non-suppurative meningoencephalitis (*n =* 7) Bronchointerstitial pneumonia (*n =* 4)	Calabria (*n =* 10), Campania (*n =* 5), Sicily (*n =* 3), Tuscany (*n =* 2), Puglia (*n =* 1), and Sardinia (*n =* 1)	(28)
2021	93/217	26.88% (25/93)	*Stenella coeruleoalba*: *n =* 14 *Tursiops truncatus*: *n =* 9 *Balaenoptera physalus*: *n =* 1 *Grampus griseus*: *n =* 1	Undetermined *n =* 1 Neonates-calves: *n =* 3 Juveniles-subadults: *n =* 8 Adults: *n =* 13	*n =* 11 (11/17, 64.70%) Non-suppurative meningoencephalitis (*n =* 7) Bronchointerstitial pneumonia (*n =* 5) Lymphoid depletion (*n =* 3)	Campania (*n =* 9), Liguria (*n =* 5), Calabria (*n =* 4), Tuscany (*n =* 4), Sardinia (*n =* 1), Sicily (*n =* 1), and Lazio (*n =* 1)	(29)
**Total**	**354/829**	**31.92% (113/354)**	***Stenella coeruleoalba*****:** ***n** **=*** **80 (80/113, 70.80%)** ***Tursiops truncatus*****:** ***n** **=*** **20 (20/113, 17.70%)** ***Physeter macrocephalus*****:** ***n** **=*** **6 (6/113, 5.31%)** ***Balaenoptera physalus*****:** ***n** **=*** **3 (3/113, 2.66%)** ***Grampus griseus*****:** ***n** **=*** **2 (2/113, 1.77%)** ***Globicephala melas*****:** ***n** **=*** **1 (1/113, 0.88%)** ***Ziphius cavirostris*****:** ***n** **=*** **1 (1/113, 0.88%)**	**Undetermined** ***n** **=*** **1(1/113, 0.88%) Neonates-calves:** ***n** **=*** **8 (8/113, 7.08%) Juveniles-subadults:** ***n** **=*** **47 (47/113, 41.59%) Adults:** ***n** **=*** **57 (57/113, 50.44%)**	***n** **=*** **56 (56/87, 64.36%) Non-suppurative meningoencephalitis (*****n** **=*** **39) Bronchointerstitial pneumonia (*****n** **=*** **19) Lymphoid depletion (*****n** **=*** **12)**	**Calabria (*****n** **=*** **26), Campania (*****n** **=*** **25), Liguria (*****n** **=*** **15), Tuscany (*****n** **=*** **12), Sardinia (*****n** **=*** **11), Sicily (*****n** **=*** **10), Puglia (*****n** **=*** **6), Lazio (*****n** **=*** **5), Abruzzo (*****n** **=*** **2), and Molise (*****n** **=*** **1)**	

^*^Certain CeMV-positive animals exhibited multiple compatible lesions.

The species tested in our study included striped dolphins (*n* = 198), bottlenose dolphins (*Tursiops truncatus*) (*n* = 126), sperm whales (*Physeter macrocephalus*) (*n* = 7), Risso's dolphins (*Grampus griseus*) (*n* = 7), fin whales (*Balaenoptera physalus*) (*n* = 5), long-finned pilot whales (*Globicephala melas*) (*n* = 4), short-beaked common dolphins (*Delphinus delphis*) (*n* = 4), Cuvier's Beaked whales (*Ziphius cavirostris*) (*n* = 2), and a false killer whale (*Pseudorca crassidens*) (*n* = 1). Molecular positivity was found mainly in striped dolphins (80/113, 70.80%), followed by bottlenose dolphins (20/113, 17.70%), sperm whales (6/113, 5.31%), fin whales (3/113, 2.66%), Risso's dolphins (2/113, 1.77%), long-finned pilot whales (1/113, 0.88%), and a Cuvier's beaked whale (1/113, 0.88%) (Table 1). Notably, all false killer whales and common dolphins tested negative for the presence of CeMV.

A histopathological study was performed on 87 CeMV-positive individuals (87/113, 76.99%), of whom 64.36% (56/87) presented lesions compatible with DMV infection (Table 1). The main lesions detected were non-suppurative meningoencephalitis (*n* = 39), bronchointerstitial pneumonia (*n* = 19), and lymphoid depletion (*n* = 12). With regard to those without CeMV-associated lesions (*n* = 31), 15 (15/31, 48.38%) presented coinfections with viral [Herpesvirus (*n* = 6)], bacterial [*Brucella* spp. (*n* = 4), *Vibrio* spp. (*n* = 2), *Salmonella* 1,4,[5],12:i:- (*n* = 1) (39), *Rhodococcus* spp. (*n* = 1), *Klebsiella* spp. [*n* = 1], and *Erysipelothrix rhusiopathiae* (*n* = 1)], or protozoan [*Toxoplasma gondii* (*n* = 2)] pathogens, and/or severe nematode infestations (*n* = 3) (26–29). Out of these 31 CeMV-positive animals without associated lesions, the majority were subadults (*n* = 14) or adults (*n* = 13), mainly striped dolphins (*n* = 22), and there were no differences in terms of sex among them.

Phylogenetic analysis was performed based on good-quality sequences obtained from 24 individuals, including striped dolphins (19/24, 79.16%), bottlenose dolphins (4/24, 16.67%), and sperm whales (1/24, 4.17%) (Table 2). The results of the CeMV strain characterization, along with biological and epidemiological data of the individuals analyzed, are summarized in Table 2.

**Table 2:** Result of the molecular analysis of the 24 animals on which a characterization was carried out, and their epidemiological information.

**ID**	**Ref. lab**	**DCC**	**NS**	**Species**	**Age**	**Sex**	**SD**	**Region**	**AG**	**Tissues tested**	**References**
1	2926	2	Moderate	Sc	J	M	13/01/2018	Liguria	N (OP751905), P (OP765378)	Brain, lung, liver, blood plasma, pulmonary lymph node, prescapular lymph node, spleen, kidney, urinary bladder, cerebrospinal fluid, **pharyngeal tonsils**, skin lesion, and endoparasite	(40)
2	5129	4	Poor	Sc	A	M	20/01/2018	Liguria	N (OP751904), P (OP765377)	Brain, **lung**, liver, blood, prescapular lymph node, pulmonary lymph node, spleen, kidney, and urinary bladder	
3	41779	1	Good	Sc	A	M	17/04/2018	Calabria	F (OP751884), N (OP751908), P (OP765381)	**Brain**	(40)
4	62728	2	Poor	Sc	A	M	26/06/2018	Calabria	F (OP751901), N (OP751922), P (OP765396)	**Brain**	(40, 41)
5	87558	2	Moderate	Sc	C	Fe	29/10/2018	Liguria	F (OP751891), N (OP751913), P (OP765386)	Brain, **lung**, liver, prescapular lymph node, pulmonary lymph node, spleen, **pharyngeal tonsils**, and urinary bladder	(40)
6	100927	1	Good	Sc	A	M	19/11/2018	Calabria	F (OP751902), N (OP751923), P (OP765397)	**Brain**	(40)
7	619	2	Poor	Sc	A	M	03/01/2019	Liguria	F (OP751885), N (OP751909), P (OP765382)	Brain, lung, liver, prescapular lymph node, **pulmonary lymph node**, pharyngeal tonsils, tongue, urinary bladder, spleen, and kidney	(40)
8	9553	1	Good	Sc	J	Fe	09/01/2019	Calabria	F (OP751892), N (OP751914), P (OP765387)	**Brain**	(40)
9	4561	5	Poor	Sc	A	ND	17/01/2019	Liguria	F (OP751886), N (OP751910), P (OP765383)	**Brain**, prescapular lymph node, mesenteric lymph node, and kidney	
10	23352	2	Good	Sc	A	Fe	27/01/2019	Campania	F (OP751889)	**Brain**	(40)
11	25089	2	Moderate	Sc	J	M	29/01/2019	Calabria	F (OP751890), N (OP751912), P (OP765385)	**Brain**	(40)
12	23336	3	Poor	Sc	A	M	08/02/2019	Campania	F (OP751888)	**Brain**	(40)
13	21724	2	Good	Sc	A	Fe	05/03/2019	Liguria	F (OP751887), N (OP751911), P (OP765384)	**Brain**, lung, liver, prescapular lymph node, tracheobronchial lymph node, pulmonary lymph node, spleen, kidney, pharyngeal tonsils, urinary bladder, and placenta	(40)
14	42472	4	Moderate	Tt	A	M	07/05/2019	Liguria	F (OP751893), P (OP765388)	Brain, **lung**, liver, prescapular lymph node, mesenteric lymph node, spleen, kidney, and urinary bladder	
15	59948	4	Moderate	Pm	J	M	21/05/2019	Sicilia	N (OP751916), P (OP765390)	Brain, **lung**, prescapular lymph node, pulmonary lymph node, and spleen	
16	59260	2	Moderate	Tt	A	M	04/07/2019	Liguria	F (OP751895), N (OP751915), P (OP765389)	Brain, **lung**, liver, prescapular lymph node, tracheobronchial lymph node, mesenteric lymph node, spleen, kidney, and pharyngeal tonsils	(40)
17	62877	3	Moderate	Sc	A	M	21/07/2019	Liguria	F (OP751894)	Brain, lung, liver, **prescapular lymph node**, pulmonary lymph node, mesenteric lymph node, spleen, and kidney	
18	38375	3	Good	Sc	J	M	22/04/2020	Calabria	N (OP751917), P (OP765391)	**Brain**	
19	85828	3	Moderate	Sc	A	Fe	17/06/2020	Sicilia	F (OP751896), N (OP751918), P (OP765392)	**Brain**	
20	85829	3	Moderate	Sc	A	M	17/06/2020	Sicilia	F (OP751897), N (OP751919), P (OP765393)	**Pulmonary lymph node**	
21	85830	3	Good	Sc	A	M	07/07/2020	Sicilia	F (OP751898), N (OP751920), P (OP765394)	**Brain**	
22	2564	4	Good	Sc	A	M	10/12/2020	Campania	F (OP751899), P (OP765395)	**Brain**, lung, spleen, and kidney	
23	73951	3	Moderate	Tt	J	Fe	10/09/2021	Liguria	F (OP751900), N (OP751921)	Brain, **lung**, liver, prescapular lymph node, spleen, kidney, and pharyngeal tonsils	
24	177	2	Good	Tt	A	Fe	24/12/2021	Liguria	F (OP751903)	**Brain**, lung, liver, prescapular lymph node, mesenteric lymph node, spleen, kidney, pharyngeal tonsils, and urinary bladder	

Samples positive to molecular diagnosis are underlined, and samples selected for sequencing are in bold. Accession numbers of DMV sequences are indicated in parentheses. ID, identification; Ref. lab., Laboratorial reference; DCC, decomposition condition code; NS, nutritional status; AG, amplificated genes; Sc, Stenella coeruleoalba; Tt, Tursiops truncatus; Pm, Physeter macrocephalus; J, juvenile-subadult; A, adult; C, calf-neonate; M, male; Fe, female; F, fusion protein gene; N, nucleoprotein gene; P, phosphoprotein gene.

A total of 57 sequences were identified and submitted to GenBank under accession numbers OP751884-OP751905, OP751908-OP751923, OP765377, OP765378, and OP765381-OP765397 (Table 2).

**Figure 2:**
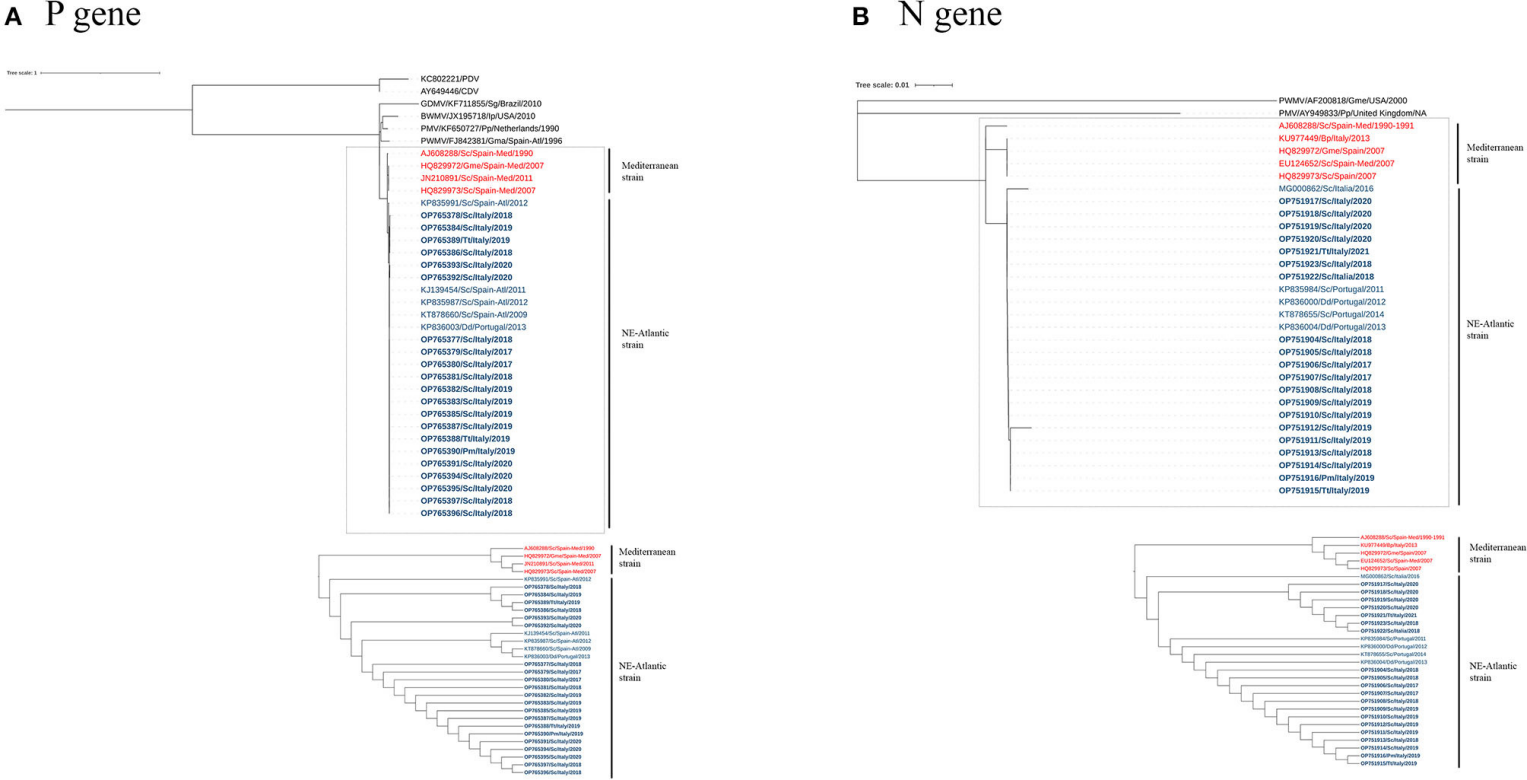
A maximum-likelihood phylogram of CeMV, based on the partial nucleotide sequence of the P **(A)** and N **(B)** genes. Dotted lines highlight the DMV sequences, with the Mediterranean and NE-Atlantic strains. The bottom image features an ignoring branch length cladogram, providing a detailed observation of the two groups' distinct separation as expected. CeMV nucleotide sequences are named according to accession number, host species, collection place, and collection date. Sequences identified in this study have been highlighted in bold. The DMV sequences from Mediterranean and NE-Atlantic strains have been distinguished using the colors red and blue, respectively. CDV, Canine Distemper Virus; PDV, Phocine Distemper Virus; GDMV, Guiana Dolphin Morbillivirus; BWMV, Beaked Whale Morbillivirus; PMV, Porpoise Morbillivirus; PWMV, Pilot Whale Morbillivirus; Sc, *Stenella coeruleoalba*; Pm, *Physeter macrocephalus*; Tt, *Tursiops truncatus*; Dd, *Delphinus delphis*; Gma, *Globicephala macrorhynchus*; Gme, *Globicephala melas*; Ip, *Indopacetus pacificus*; Pp, *Phocoena*; Sg, *Sotalia guianensis*; Spain-Med, Spanish Mediterranean coast; Spain-Atl, Spanish Atlantic coast.

The phylogeny constructed based on the sequences of the P and N genes showed two main clades in the DMV sequences that separate the Mediterranean and NE-Atlantic strains (Figure 2), and the sequences of this study clustered together with the latter. Accordingly, our sequences showed the highest identity with GenBank sequences MF472881, MG000861, MF472882, KP835987, KP835991, KP836003, and KT878660 (identities: 1.0000 — 0.9908) for the P gene and with GenBank sequences MG000862, KP835984, KP836000, KT878655, and KP836004 (identities: 1.0000 — 0.9943) for the N gene (Supplementary material), which are considered NE-Atlantic strains.

## Discussion

Our study assessed the prevalence of CeMV in cetaceans stranded along the Italian coasts between 2018 and 2021 and successfully molecularly typed the detected isolates. These insights enhance our knowledge on the current epidemiological status of CeMV in the Mediterranean Sea.

Various prevalence rates of CeMV were reported in different locations and species worldwide during periods that were not associated with unusual mortality events (UMEs). These rates include 1.81% (1/55) in stranded beaked whales from the Canary Islands (42), 4.25% (2/47) in humpback whales' (*Megaptera novaeangliae*) exhaled breath in Brazil (43), 5.73% (16/279) in cetaceans stranded along the Portuguese and Galician (Spain) coasts (38), 14.61% (13/89) in cetaceans stranded on the Mediterranean Spanish coast (Catalonia) (44), 16.66% (2/12) in Risso's dolphins (*Grampus griseus*) from the Canary Islands (45), 19.68% (37/188) in stranded cetaceans from Italy (40), 21.74% (5/23) in fin whales (*Balaenoptera physalus*) from Italy (19), 24.19% (15/62) in cetaceans stranded in Hawaii (3), 27.50% (11/40) in stranded cetaceans from Brazilian waters (46), and 60.0% (3/5) in southern right whales (*Eubalaena australis*) from Brazil (47). In the present study, we report a CeMV prevalence of 31.9% (113/354) between 2018 and 2021 in Italian waters, with a peak in 2019 (45.3%) and the lowest prevalence in 2020 (23.9%) (Table 1). These CeMV prevalences are among the highest reported without an association with an outbreak.

Molecular positivity for CeMV was detected in cetaceans stranded in all the marine sectors of the Italian coastline, with the only exception being the northern Adriatic coast (*n* = 0 in the regions of Emilia-Romagna, Veneto, and Friuli-Venezia Giulia) (Table 1 and Figure 1). This could be related to the fact that most of the cetaceans stranded during this period in the aforementioned regions (56/70, 80.0%) had a DCC of 4 or 5 (26–29), which might cause the degradation of the RNA virus. However, the area where the highest number of CeMV-positive cetaceans stranded during the study period was the southwest of the Italian peninsula (Calabria, *n* = 26; Campania, *n* = 25) (Table 1 and Figure 1). It has been suggested that chemical contaminants have the potential to impact susceptibility to CeMV infection (48). A recent investigation has revealed a correlation between the presence of pathological evidence of infection and the elevated levels of chemical pollutants in the Pelagos Sanctuary, located in northern Italy (49). Considering the existing body of research indicating higher concentrations of pollutants in southern Italy (41, 50), it is plausible to attribute the higher number of CeMV-positive animals in this geographical region to such environmental factors. However, further studies are necessary to elucidate the origins of these observed differences in prevalence between the different regions. Continued monitoring of strandings and their causes will provide valuable insights into whether these geographical patterns are repeated and help identify potential underlying causes.

Regarding the DMV strains present in the Mediterranean Sea, recent evidence indicates that the previous DMV Mediterranean strain has been replaced by the NE-Atlantic strain (18, 21, 22), first reported in the Mediterranean Sea in 2012 (20). P and N gene sequences described in the present study clustered together with NE-Atlantic DMV strain sequences (Figure 2). These sequences were identical or showed the highest identity with the NE-Atlantic strain, detected on the Portuguese and Spanish Atlantic coasts and in Italy (see Supplementary material). Taken together, these results provide evidence of an active circulation of the NE-Atlantic strain along the Italian coastline during the studied period, as previously reported (18, 21, 22, 40).

Histopathological analysis revealed lesions compatible with CeMV infection in 64.36% (56/87) of the cetaceans analyzed (Table 1). The main lesions detected were non-suppurative meningoencephalitis, bronchointerstitial pneumonia, and lymphoid depletion (Table 1), in agreement with the literature (2, 14). The neurological lesions associated with CeMV in some of these animals (ID1, ID3, ID4, ID5, ID6, ID7, ID8, ID10, ID11, ID12, ID13, and ID16) have been recently characterized and classified as acute or subacute (40).

It was hypothesized that the epidemiology of the DMV strain that circulated in 2011 along the Spanish Mediterranean coast was changing from an epizootic to an enzootic infection (14). Similarly, chronic forms of DMV infection were predominant in 2011 (51) and 2013 (16) in Spain and Italy, respectively. However, during the 2016 Italian UME, acute and subacute forms of DMV infection were mainly reported (18, 52). This could be explained by the fact that the cetacean population of the Mediterranean Sea was naïve to this NE-Atlantic strain (18, 20, 22) and the greater neurovirulence described in this strain, which prevented affected cetaceans from surviving the acute and subacute stages of the infection, as previously proposed (40). In line with this, non-suppurative meningoencephalitis was the main lesion detected in this series of cases (Table 1). However, the present findings have demonstrated that, in more than a third (*n* = 31/87, 35.63%) of the 87 CeMV-positive individuals that received a histopathological examination, no lesions consistent with DMV infection were detected, suggesting a chronic form of the infection (no lesions directly attributable to CeMV, but viral antigen is detectable) (2). In addition, almost half of these animals (*n* = 14/31) presented coinfections with important viral, bacterial, or protozoan agents, and/or severe nematode infestations (26–29, 39, 53). These coinfections are typical of the chronic phase of DMV infection and are related to host immunosuppression caused by the virus (2). However, a complete and exhaustive histopathological study was not always performable due to the autolysis status of the animals involved: only 12 of these 31 animals were fresh carcasses (DCC = 2), 11 were in moderate decomposition (DCC = 3), and eight in advanced decomposition (DCC = 4) (data not shown). Therefore, it is possible that our ratio of CeMV-positive cetaceans lacking CeMV-associated lesions was overestimated due to the conservation status of the animals, which in some cases precluded a reliable examination. This could explain the difference in terms of the predominant stage of infection reported in a recent study (40) involving some of the animals described here (acute/subacute vs. chronic). In addition, in the aforementioned study, the stage of infection was determined by a multiparametric assessment from the cerebral and cerebellar cortex samples (40), while in our study, a systemic determination was considered.

The infection with the NE-Atlantic strain was considered endemic in the striped dolphin populations from Portugal and Galicia since no outbreaks were detected and positive samples were detected annually, indicating that the virus was actively circulating (38). In addition, the prevalence obtained in that study was 24% (38), which is very similar to the prevalence obtained in the last 2 years of our study: 23.91% (2020) and 26.88% (2021). Thus, our results support the possibility of an endemic circulation of this strain in the Mediterranean Sea, as previously theorized (21).

Approximately half (57/113, 50.44%) of the CeMV-positive cetaceans stranded in Italy during this period were adults, while the others were juveniles-subadults (41.59%, 47/113), neonates-calves (7.08%, 8/113), and, in one case (0.88%, 1/113), the age could not be determined (26–29). However, our results have demonstrated that most of the CeMV sequences were obtained from adult individuals (17/24, 70.83%), while the rest were obtained from juveniles-subadults (6/24, 25.00%) and neonates-calves (1/24, 4.17%) (Table 2). In contrast, Giorda et al. (40) reported a slightly higher proportion of CeMV-positive juveniles-subadults (54.8%) than adults (45.2%), and no CeMV-positive neonates-calves. These observations differ from the events reported during the latest Mediterranean UMEs, which involved mainly young animals (newborns, juveniles, and subadults), probably due to a lack of specific antiviral immunity (14, 16).

This NE-Atlantic strain most likely entered the immunologically naïve striped dolphin population with a lack of specific antiviral immunity, resulting in a UME in 2016 (18) and several associated strandings since then (21, 40). However, it is possible that some individuals outgrow the acute/subacute phase of the infection, resulting in chronic forms with detectable DMV antigen but with no associated lesions. This specific immunity would explain the absence of UME since 2016. These observations are in good agreement with previous findings that support an endemic DMV circulation in the Mediterranean Sea and with the occurrence of cyclic outbreaks when herd immunity decreases (14). Nevertheless, further research is needed to clarify the epidemiology of this new DMV strain in the Mediterranean Sea.

It has been suggested that the NE-Atlantic strain entered the Mediterranean Sea through the Strait of Gibraltar, where cetacean populations from both seas come into contact, and subsequently spread into the Mediterranean Sea (18, 21, 22), where it has continued to circulate until 2021, as this study confirms. As previously hypothesized, for the possibility of an endemic circulation of CeMV in the Mediterranean Sea, the transmission and maintenance of CeMV strains between different species should be considered (21). In the Mediterranean Sea, the striped dolphin is the species in which CeMV has been detected most frequently (11, 13, 14, 16, 18, 20–22). Accordingly, 70.80% (80/113) of the CeMV-positive cetaceans stranded in Italy during the study period were striped dolphins (Table 1). However, in the literature, it has also been reported in the Mediterranean Sea in other species such as short-beaked common dolphins (18), bottlenose dolphins (15, 22), long-finned pilot whales (54), Cuvier's beaked whales (55), sperm whales (17, 22), and fin whales (19, 22). In line with this, between 2018 and 2021 in Italy, CeMV was detected in bottlenose dolphins, fin whales, long-finned pilot whales, Cuvier's beaked whales, and sperm whales (Table 1). Some of these species, such as long-finned pilot whales, Cuvier's beaked whales, and sperm whales, have been suggested to spread the virus between geographically distant populations (42, 54, 56). Specifically, sperm whales are known to be susceptible to infection by the NE-Atlantic CeMV strain (22), and they have been suggested to be a spillover host with a role in disseminating CeMV to cetaceans from widely dispersed geographical regions since they travel long distances during seasonal migration (56). During the period spanning from 2018 to 2021, a total of six sperm whales tested positive for CeMV, as indicated in Table 1. Among these cases, the presence of the NE-Atlantic strain of CeMV was confirmed in one individual (Table 2). However, the NE-Atlantic CeMV strain was also previously reported in bottlenose dolphins in the Mediterranean Sea (40). During the study period, this strain was reported in 20 bottlenose dolphins (Table 1), with at least four of these individuals found to be infected with the NE-Atlantic strain (Table 2). However, the epidemiological role of these species in the transmission pattern of this strain in the Mediterranean Sea is still unknown, and further studies would be necessary to shed light on this field.

## Conclusion

In this study, we report a CeMV prevalence of 31.9% in cetaceans stranded along the Italian coasts between 2018 and 2021. The greater proportion of CeMV-positive cetaceans during the study period was found in the south-west of the Italian peninsula (Figure 1). A total of 57 sequences from novel strains were generated and submitted to GenBank. Phylogenetic analysis revealed that these sequences belonged to the NE-Atlantic DMV strain. The species affected by the detected strains were striped dolphins, bottlenose dolphins, and sperm whales, and the individuals were mainly adults and, to a lesser extent, juveniles-subadults and neonates-calves. The evidence from this research points toward the idea of an endemic circulation of the NE-Atlantic strain in the Italian Sea based on the epidemiological, phylogenetic, and histopathological information.

## Data availability statement

The datasets presented in this study can be found in online repositories. The names of the repository/repositories and accession number(s) can be found in the article/Supplementary material.

## Ethics statement

Ethical review and approval was not required for the animal study because in the collection of post-mortem tissues for research purposes, the approval of the corresponding Ethics Committee is not required.

## Author contributions

Conceptualization: FG, CG, and IV-C. Data curation: VM and CG. Funding acquisition: CC and CG. Investigations: SP, MG, LS, KV, RP, FDN, GL, PA, FG, and CG. Methodology: FG and CG. Software: IV-C and SP. Supervision: SP, PA, JMS-V, FG, and CC. Writing—original draft: IV-C. Writing—review and editing: FG, CG, and SP. All authors contributed to the article and approved the submitted version.
